# Ndfip1 restricts Th17 cell potency by limiting lineage stability and proinflammatory cytokine production

**DOI:** 10.1038/srep39649

**Published:** 2017-01-04

**Authors:** Awo Akosua Kesewa Layman, Stephanie L. Sprout, Dylan Phillips, Paula M. Oliver

**Affiliations:** 1 Medical Scientist Training Program, Perelman School of Medicine at the University of Pennsylvania, 3400 Civic Center Boulevard, Building 421, Philadelphia, PA 19104, USA; 2 Biomedical graduate Studies, Immunology Graduate Group, 357 Biomedical Research Building II/III, 421 Curie Boulevard, University of Pennsylvania, Philadelphia, PA 19104, USA; 3 The Children’s Hospital of Philadelphia, Cell Pathology Division, 3401 Civic Center Boulevard, Philadelphia, PA 19104, USA.; 4 Department of Pathology and Laboratory Medicine, Perelman School of Medicine at the University of Pennsylvania, 3400 Civic Center Boulevard, Building 421, Philadelphia, PA 19104, USA.

## Abstract

While Th17 cells can protect against colonization by pathogenic organisms, they also have the potential to become pathogenic and promote autoimmune and inflammatory diseases. Mechanisms that control their pathogenic potential remain poorly understood. Here we show that Ndfip1, a co-activator of the E3 ubiquitin ligase Itch, restricts the frequency and pathogenicity of Th17 cells. Mice lacking Ndfip1 have increased numbers of Th17 cells, and this increase is cell intrinsic. We found that Ndfip1 restricts production of the proinflammatory cytokines in Th17 cells. Increased cytokine production correlated with reduced degradation and accumulation of RORγT. When transferred *in vivo*, Th17 cells lacking Ndfip1 were more likely to maintain their ability to make IL-17, were more potent proinflammatory cytokine producers, and were powerful inducers of colitis. Together our data support an essential role for Ndfip1 in degrading RORγT and suppressing Th17 lineage stability, proinflammatory cytokine production, and pathogenicity.

Since their discovery over a decade ago, Th17 cells, or CD4+ T cells producing the signature cytokine IL-17A, have been determined to play an important role in defense against fungal and extracellular pathogens^1^,^2^,^3^. Th17 cells express the transcription factors RORγT^4^, RORα^5^ and STAT3^6^,^7^ and can secrete several additional cytokines including IL-17F, IL-22, IFNγ and TNFα. Humans bearing mutations that prevent Th17 cell differentiation or IL-17 receptor signaling, are susceptible to fatal candidiasis^8^,^9^,^10^,^11^ indicating the importance of Th17 cells in anti-fungal immunity. On the other hand, increased numbers of Th17 cells in certain inflammatory disorders have been correlated with disease severity. This includes conditions such as Crohn’s disease, multiple sclerosis, rheumatoid arthritis, psoriasis and psoriatic arthritis. Blocking antibodies that target IL-17A or IL-23, a cytokine that increases Th17 cell numbers, have been used in clinical trials for these diseases, with mixed results^12^,^13^. In some cases, treatment has led to increased susceptibility to infection^14^,^15^,^16^. Thus, identifying ways to adjust the potency of Th17 cell pathogenicity may be of therapeutic benefit.

In most cases, it is not known how the Th17 cells that drive autoinflammatory diseases develop; whether they develop from previously functional Th17 cells that become dysregulated or whether they can be identified *ab initio* based on unique characteristics. In support of the hypothesis that pathogenic Th17 cells may be programmed to be pathogenic at their induction, it is thought that the inflammatory conditions under which a Th17 cells is generated may affect its pathogenicity. Related to this, some factors that are reported to influence the pathogenic potency of Th17 cells include their exposure to IL-23 during differentiation. Such exposure results in the formation of a complex that contains the transcription factors Blimp1, RORγT, STAT3, p300, HIF1α, BATF and IRF4. Together, these factors cooperate to drive the expression of genes such as *il17a, il23r* and *csf2*^8^,^17^. Furthermore, pathogenic Th17s have been reported to show a gene profile that includes *tbx21, csf2* and *ccl5* (Rantes), among others^18^. Csf2-driven GM-CSF production in particular is thought to be important for the pathogenicity of Th17 cells, especially in disease models such as Experimental Autoimmune Encephalomyelitis (EAE)^19^,^20^. IFNγ expression by Th17 cells, which can be induced by IL-23 signaling and/or high levels of *Tbx21*, is also associated with EAE and colitis in models of Inflammatory Bowel Disease (IBD)^21^,^22^. Defining mechanisms that restrict these factors and thus limit Th17 cell pathogenicity could aid the development of therapeutics that could reduce the pathogenic potency of Th17 cells.

Ubiquitylation of transcription factors and lineage-defining proteins is one under-appreciated means of regulating the differentiation and function of T helper subsets, including Th17 cells^23^. Surprisingly little is known about how ubiquitylation of proteins affects the generation of pathogenic versus non-pathogenic Th17 cells. Ubiquitylation is a post-translation modification that results in the addition of ubiquitin to a protein substrate within the cell. This process involves the concerted action of three main enzymes: an E1 ubiquitin activating enzyme, an E2 ubiquitin conjugating enzyme, and an E3 ubiquitin ligase. E3 ligases can determine substrate specificity as well as the type of ubiquitin chain placed on the substrate, and thus determine the ultimate fate of the substrate.

Some E3 ubiquitin ligases require accessory proteins to activate their catalytic function. Itch is one example of this. Nedd4-family interacting protein 1 (Ndfip1) is a protein that activates the catalytic function of members of the Nedd4 family. Mice deficient in Ndfip1 protein develop a spontaneous inflammatory disease marked by an increase in the frequency of several subsets of CD4+ T cells including CD44+ T cells^24^,^25^,^26^, IL-17A+ Th17 cells^27^, IFNγ+ CD4 T cells^26^,^28^ and IL-4+ Th2 cells^24^,^28^,^29^ at mucosal barrier surfaces such as the lungs, GI tract, and the skin. The predominant defect observed in these mice –namely the increase in Th2 cells- occurs because Ndfip1 is not available to activate Itch, an E3 ligase that is required for polyubiquitylation and degradation of the transcription factor JunB. Thus, in activated Ndfip1- or Itch-deficient T cells, JunB protein levels accumulate, driving increased IL-4 production and a consequent rise in the numbers of differentiating Th2 cells.

Ndfip1 has been indirectly implicated in the regulation of Th17 cells. It has been shown that excessive IL-4 production from Ndfip1-deficient Th2 cells drives tissue damage and eosinophilia, which in turn leads to an increase in secreted factors such as IL-6, and favors *in vivo* Th17 generation^27^. However, it is unknown whether Ndfip1 has direct roles within Th17s. Very recently, the catalytic E3 ligase, Itch, was shown to ubiquitylate RORγT, driving its degradation and helping to limit the generation of Th17 cells in the colon^30^. However, it remains unclear how the increased levels of RORγT that occur in the absence of Itch impact Th17 cell function.

In this study, we show that Ndfip1 or Itch E3 ligase deficiency drives an increase in Th17 cell numbers at barrier surfaces. Increased Th17 cell abundance in Itch- and Ndfip1-deficient animals does not depend on the well-characterized roles for these two proteins in T cell activation or in IL-4-mediated inflammation. Ndfip1 and Itch do not control the numbers of cells differentiating into Th17 cells *in vitro*, rather they limit the production of cytokines by the cells following differentiation. As with their Itch-deficient counterparts, Ndfip1-deficient Th17 cells are unable to degrade RORγT upon T cell stimulation, and increased RORγT correlated with an elevated production of the proinflammatory cytokines IL-17A, IFNγ or GM-CSF. *In vivo*, Th17 cells lacking Ndfip1 were more likely than their WT counterparts to maintain lineage commitment, based on production of IL-17A, were much more likely to co-produce other pathogenic cytokines, and caused more severe colitis. Thus, Th17 cells require Ndfip1 and Itch to limit RORγT levels and prevent Th17 cells from causing severe tissue inflammation and inflammatory bowel disease.

## Results

### Loss of Ndfip1 results in a T cell-intrinsic increase in Th17 cell numbers

Naïve CD4 T cells differentiate into Th17 cells at mucosal surfaces such as the lung and colon in response to TCR stimulation, co-stimulation, and the availability of lineage-instructing cytokines including TGFβ, IL-6, IL-1β and IL-23^31^,^32^,^33^. Downstream of these signals, proteins such as RORγT, BATF and STAT3 are produced to drive the transcription and translation of proteins that are needed for establishing and maintaining Th17 identity^8^.

**Figure 1:**
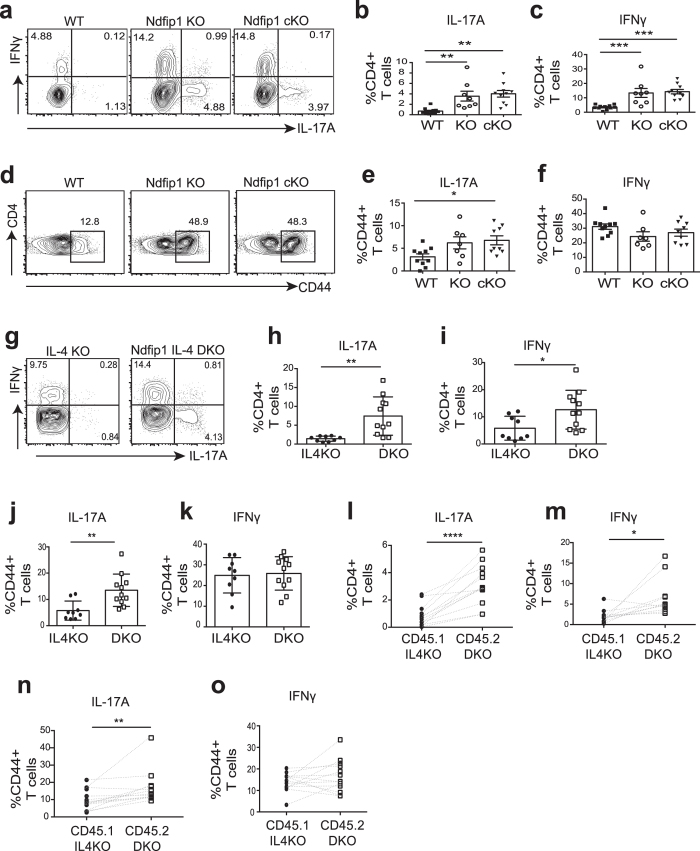
Ndfip1 limits the abundance of Th17 cells. (**a**–**f**) Flow cytometric analysis of CD3^+^ CD4+ T cells among cells isolated from lungs of wild type (WT), Ndfip1 knockout (KO), or Ndfip1^fl/fl^ CD4-Cre (cKO) animals, showing representative (**a**,**d)** or combined (**b**,**c**,**e**,**f**) data from multiple experiments. (**a**–**c**) Percentages of lung CD4+ T cells that are IL-17A^+^ (**a**,**b**) or IFNγ^+^ **(a**,**c**). (**d**) Percentages of previously activated (CD44^+^) CD4+ T cells in the lung. (**e**–**f**) Percentages of CD44^+^ CD4^+^ cells that are IL-17A+ (**e**) or IFNγ+ (**f**). (**g**–**k**) Flow cytometric analysis of CD3^+^ CD4+ T cells from lungs of Ndfip1^−/−^ IL-4^−/−^ (DKO) mice or IL-4^−/−^ (IL-4KO) controls, showing representative (**g**) or combined (**h**–**k**) data from multiple experiments. (**g**–**i**) Percentages of lung IL-17A^+^ (**g**,**h**) or IFNγ^+^ (**g**,**i**) CD4+ T cells. (**j**,**k**) Percentages of previously activated cells (gated as in panel d) that produce IL-17A^+^ (**j**) or IFNγ^+^ (**k**). (**l**–**o**) Flow cytometric analysis of CD3^+^ CD4+ T cells from lungs of CD45.1 IL-4^−/−^ (IL-4KO) and CD45.2 Ndfip1^−/−^ IL-4^−/−^ (DKO) mixed chimeras. (**l,m**) Percentages of lung IL-17A^+^ (**l**) or IFNγ^+^ (**m**) producing cells of CD45.2 DKO or CD45.1 IL-4KO origin. (**n**–**o**) Percentages of IL-17A^+^ (**n**) or IFNγ^+^ (**o**) producing cells among previously activated T cells of CD45.2 DKO or CD45.1 IL-4KO origin. In (**a**–**f**) n = 8–11 animals per genotype, analyzed at 7–9 weeks of age in 7 independent experiments using one-way ANOVA and Holm-Sidak’s multiple comparisons test. In (**g**–**k**) n = 9–11 animals per genotype analyzed at 6–16 weeks old in 4 independent experiments using an unpaired T test. In (**l**–**o**) n = 13 mixed chimera animals at 7–8 weeks old in 2 independent experiments using a paired T test. *p < 0.05, **p < 0.01, ***p < 0.001, ****p < 0.0001. All error bars represent mean +/− SEM.

In Ndfip1-deficient animals, tissue destruction mediated by potent IL-4 producing Th2 cells has been shown to cause increased IL-6 at tissue sites, and this is thought to drive an increase in mucosal Th17 generation *in vivo*^34^. However, it is also possible that this increase in Th17 cells may be due to a loss of Ndfip1 within the T cell lineage. To investigate how Ndfip1 functions within CD4 T cells to limit the abundance of Th17 cells, we first compared the Th17 cell (CD4+ IL-17A+) frequency and number in mice which constitutively lack Ndfip1 to those that lack Ndfip1 only in T cells (Ndfip1fl/fl CD4-Cre, referred to as cKO mice). Consistent with previously published data, we found an increase in the frequency of Th17 cells in the lungs (Fig. 1a and b) of mice constitutively lacking Ndfip1 over WT controls. Importantly, with T cell-specific deletion of Ndfip1, we found similarly increased numbers of Th17 cells. An increase in IFNγ+ CD4 T cells in the lungs has also been described^26^, supporting the possibility that Ndfip1 regulates IFNγ+ and IL-17A+ cells via a similar mechanism. Consistent with this, both Ndfip1-deficient animals, and mice with a T cell-specific deletion in Ndfip1, also showed an increase in IFNγ+ CD4 T cells in the lungs compared to the WT controls (Fig. 1c). These data reveal that a loss of Ndfip1 in T cells can lead to an increase in numbers of both Th17 and Th1 cells.

Mice that lack Ndfip1 constitutively, or in the T cell compartment, contain high numbers of CD44+ CD4+ T cells^24^,^25^,^26^. In order to become a Th17 cell, a naïve CD4 T cell must first become an activated, CD44+ cell. Thus, we next tested whether the increase in Th17 cells was due to increased T cell activation. We gated exclusively on CD44+ CD4 T cells (Fig. 1d) and then determined frequency of IL-17A+ or of IFNγ+ cells. For Th17 cells, even after limiting our analysis to CD44+ cells, Ndfip1-deficient T cells were significantly more likely to produce IL-17A (Fig. 1e). In contrast, when we gated in this manner, WT and Ndfip1-deficient T cells were somewhat less likely to be IFNγ+ Th1 cells, but this difference was not significant (Fig. 1f). To test whether the variability of Th17 cell numbers in Fig. 1a–f was influenced by differences in cage-to-cage microbial communities, we re-examined the data in Fig. 1a–f, focusing only on cage-matched littermates. We found deficiency of Ndfip1 resulted in a significant increase in Th17 cells regardless of whether we compared animals to controls housed in the same or different cages and regardless of whether the animals came from the same, or different, mothers (Supplementary Fig. S1). Taken together these data support that Ndfip1 regulates both Th1 and Th17 cell numbers via limiting T cell activation. However, these data also support that Ndfip1 imposes additional restrictions that selectively limit Th17 cell abundance.

Ndfip1-deficient CD4+ T cells secrete excess IL-4^24^,^28^,^29^ and this can impact the differentiation of alternate cell fates or affect the activation state of T cells. For Th17 cells, IL-4 has been shown to both promote or inhibit their differentiation^27^,^31^,^33^,^35^. To determine whether IL-4 was responsible for the increased Th17 cell frequencies in Ndfip1-deficient animals, we analyzed Th17 cells in mice that lack both IL-4 and Ndfip1 (Ndfip1 IL-4 DKO animals). In the IL-4KO controls, as in WT animals, there were very few (<1%) Th17 cells in the lungs at steady state. However, in Ndfip1 IL-4 DKO animals, we observed increased frequencies of Th17 cells (Fig. 1g and h) as well as IFNγ+ CD4 cells (Fig. 1g–i). Consistent with our prior findings, when only CD44+ CD4+ T cells were considered, DKO T cells were more likely to produce IL-17A (Fig. 1j), but there was no difference in the percentage of cells that made IFNγ+ (Fig. 1k). This indicated that Ndfip1 limits Th17 cell numbers via an IL-4 independent mechanism.

Ndfip1 restriction of Th17 cell numbers could be intrinsic, due to the loss of Ndfip1 within Th17 cells, or extrinsic, due to factors produced by Ndfip1-deficient T cells that drive *in vivo* Th17 generation. To distinguish between these two possibilities, we generated mixed chimera animals in which Ndfip1-sufficient IL-4 KO and Ndfip1-deficient DKO Th17 cells would develop in the same cytokine milieu. Even in this mixed setting, we found similar results: Ndfip1-deficient T cells were more likely to be IL-17A+ (Fig. 1l) and IFNγ+ (Fig. 1m), and while activation could not account for the increased Th17 cells (Fig. 1n), it explained the increased IFNγ+ cells (Fig. 1o). Taken together, these data support that Ndfip1 limits the numbers of Th17 cells in a T cell intrinsic manner via a mechanism that is not shared between Th1 and Th17 cells, and is independent of IL-4 mediated inflammation.

### Ndfip1 does not limit the differentiation of Th17 cells, *in vitro*

**Figure 2:**
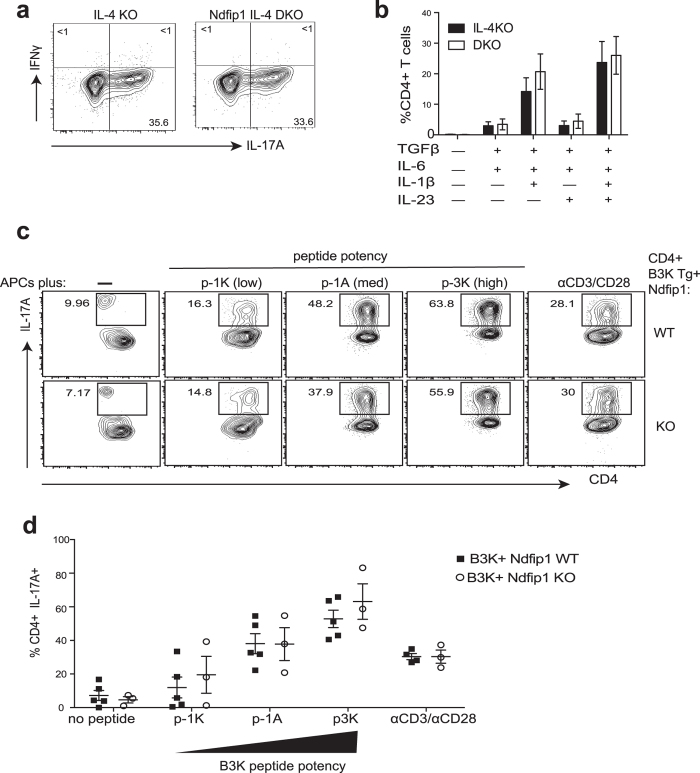
Ndfip1 does not limit the differentiation of Th17 cells *in vitro*. (**a**,**b**) Naïve sorted CD4+ T cells from IL-4 KO or Ndfip1 IL-4 DKO animals were cultured under various Th17-polarizing conditions for 5 days as noted. (**a**) Representative Th17 differentiation using IL-1β, IL-23, TGFβ, and IL-6. (**b**) Average percentages of Th17 cells generated with different combinations of TGFβ, IL-6, IL-1β and IL-23. (**c**,**d**) Peptide pulsed APCs were used to stimulate B3K transgenic T cells. T cells from Ndfip1 KO RAG1^−/−^ B3K^+^ or RAG1^−/−^ B3K^+^ control animals were incubated with APCs pulsed with peptides of low (p-1K), medium (p-1A) or high (p-3K) TCR-stimulating potency for 5 days under Th17 polarizing conditions. Cells were then restimulated and analyzed using flow cytometry. (**c**) Representative plots showing Th17 generation using no peptides, the three altered peptide ligands, or with antiCD3/anti CD28 are shown for comparison. (**d**) Combined data for multiple experiments illustrating Th17 generation, with each dot representing one mouse (**c**). In (**a**,**b**) data is shown for n = 5 animals per group in at least three independent experiments, and statistical significance was tested using a using 2-way ANOVA. Panels c-d, include data from 2 independent experiments, and the combined data was analyzed using 2-way ANOVA. All error bars represent mean +/− SEM.

The increased numbers of Th17 cells in Ndfip1-deficient animals could reflect an increase in the ability of naïve CD4 T cells to differentiate into Th17 cells or an expansion of existing Th17 cells. Thus, we examined Th17 differentiation *in vitro*. Again, to eliminate the confounding presence of IL-4, we used Ndfip1 IL-4 DKO or IL-4KO CD4+ T cells. When we differentiated Th17 cells using TGF-β, IL-6, IL-1β and IL-23, Ndfip1 IL-4 DKO cells were just as likely as controls cells to become IL17A+ cells (Fig. 2a). We then tested different mixtures of cytokines and found the same result (Fig. 2b). Next, to determine whether Ndfip1-deficient CD4 T cells were more likely than controls to become IL-17A+ cells when stimulated with a particular strength of TCR signal, we tested altered peptide ligands with B3K TCR transgenic (B3K-Tg^+^) T cells^36^. In this model, B3K-Tg^+^ animals contain CD4 T cells with a fixed T cell receptor that recognizes the foreign peptide, 3 K, in the context of I-Ab (MHC II). By creating amino acid substitutions of the 3 K peptide, altered peptides have been generated which have low, medium or high T cell activation potency^37^. We generated Ndfip1^+/+^ (WT) or Ndfip1^−/−^ B3K-Tg^+^ animals and used a low potency (p-1K), medium potency (p-1A) or high potency (p3K) peptides to stimulate the cells. We found that increasing peptide potency drove higher *in vitro* Th17 generation (Fig. 2c and d). However, Ndfip1^−/−^ and WT CD4 T cells were equally likely to become Th17s. Therefore Ndfip1 does not restrict *in vitro* Th17 differentiation.

### Ndfip1-deficient Th17 cells are more proliferative and accumulate *in vivo*

**Figure 3:**
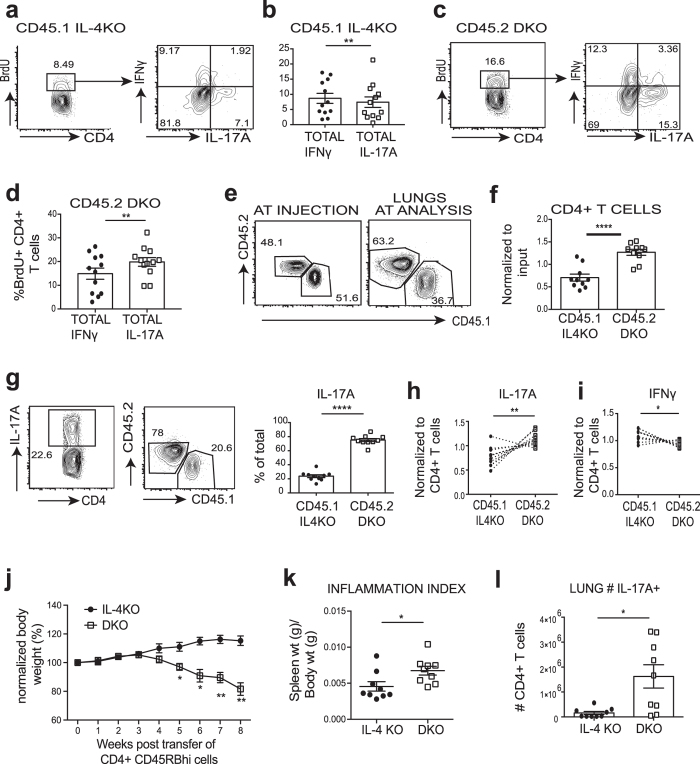
Ndfip1-deficient CD4 T cells outcompete control cells *in vivo*. (**a**–**f**) CD45.2 Ndfip1 IL-4 DKO and CD45.1 IL-4KO bone marrow was injected into RAG1^−/−^ animals and BrdU was administered 3 days prior to harvest. BrdU^+^ CD4^+^ cells in the lungs were examined by flow cytometry. (**a**) A representative plot showing the percentages of CD45.1 IL-4KO BrdU^+^ T cells in the lung as well as their production of IL-17A or IFNγ. (**b**) The percentages of IL-17A or IFNγ producing cells from multiple mice. Each dot represents a single mouse. (**c**,**d**) Same as in A and B but showing percentages for CD45.2 DKO BrdU^+^ cells in the lung. (**e**–**j**) 1:1 mixtures of naïve sorted, CD4+ T cells from Ndfip1 IL-4 DKO and IL-4KO were injected into RAG1^−/−^ animals. After 7–8 weeks, cells were isolated from the lungs, stimulated with PMA/Ionomycin, and analyzed using flow cytometry. (**e**) Percentages of CD45.2 DKO versus CD45.1 IL-4KO CD4+ T cells in the injection mixture and in the lungs at analysis. (**f**) Ratio of frequencies of CD45.2^+^ versus CD45.1^+^ CD4+ T cells in the lungs at harvest, compared to the ratio in the input injection mixture. (**g**) A representative plot showing the total Th17 cells in the lungs and the frequencies of CD45.1 or CD45.2 among Th17 cells. (**h**) Normalized ratio of percentages of congenic Th17 cells relative to CD4^+^ cells bearing each congenic marker. (**i**) The same analysis as in panel h but focused on Th1 cells. (**j**–**l**) Sorted CD4^+^ CD45RB^hi^ naïve T cells were injected into RAG−/− recipients. (**j**) Weight change relative to starting body weight. (**k**) Spleen weight normalized for body weight at harvest. (**l**) Total number of Th17 cells found in the lungs of recipients of IL-4KO or DKO T cells. In (**a**–**d**) n = 12 mixed chimera animals in 2 independent experiments. Statistical significance was calculated by paired two-tailed T tests. In (**e**–**i**) n = 11 animals in 2 independent experiments. Significance was calculated by paired T test. In (**j**–**l**) data is pooled for n = 10 IL-4KO animals and n = 9 DKO animals in 2 experiments, analysis by unpaired T test. *p < 0.05, **p < 0.01,****p < 0.0001. Error bars represent mean +/− SEM.

To understand why Ndfip1–deficient Th17 cells numbers were elevated *in vivo*, we returned to the mixed bone marrow chimera model to test Ndfip1- sufficient (CD45.1+) and -deficient (CD45.2+) Th17 cells that develop in the same cytokine environment. After reconstitution, we treated mice with 5-bromodeoxyuridine (BrdU) to assess cell cycle. Following treatment, cells were isolated from the lungs of mice and analyzed by flow cytometry. Consistent with published data^26^, we found that CD4 T cells that lack Ndfip1 were more likely than control cells to undergo proliferation *in vivo* (Fig. 3a and c). BrdU+ Ndfip1-sufficient cells in the lung were less likely to be Th17 cells (Fig. 3a and b), but BrdU+ Ndfip1-deficient cells were more likely to be Th17 cells (Fig. 3c and d). These data support that Th17 cells lacking Ndfip1 are highly proliferative.

We next used a mixed T cell-transfer model to test the capacity of Ndfip1-sufficient and -deficient Th17 cells to accumulate in a setting in which T cells of both genotypes were similarly activated. To assess this, congenically marked CD45.1 Ndfip1-sufficient and CD45.2 Ndfip1-deficient T cells (both sets of cells also lack IL-4 to eliminate any confounding effects of IL-4-driven inflammation) were transferred into Rag1−/− recipient mice. Since IL-17A and IFNγ are the predominant cytokines expressed by T cells in this system, we could examine how the loss of Ndfip1 impacts the numbers of CD4+ T cells that express these cytokines. Importantly, this was a system in which even control cells would generate a measurable population of Th17 cells so that we could better compare Ndfip1-sufficient and Ndfip1-deficient Th17s in the same environment. Naïve sorted Ndfip1-sufficient (CD45.1) and -deficient (CD45.2) CD4+ cells were mixed at a 1:1 ratio and injected into RAG1^−/−^ animals and 7 weeks after transfer, animals were analyzed. Ndfip1-deficient T cells (CD45.2+) outcompeted their Ndfip1-sufficient counterparts (CD45.1+) in the lungs (Fig. 3e and f). Furthermore, if we gated specifically on Th17 cells, we found they were much more likely to be Ndfip1-deficient (Fig. 3g) and this was true even after normalization for the differences in CD4 cell frequency (Fig. 3h). IFNγ+ cells were less likely to be Ndfip1-deficient if frequencies were normalized in this manner (Fig. 3i). Thus, Ndfip1-deficient Th17 cells are able to outnumber control cells when generated in the same setting *in vivo*.

We also performed similar transfers but instead placed Ndfip1-deficient and control T cells (both were IL-4-deficient to eliminate any effect of IL-4-mediated inflammation on Th17 cell generation *in vivo*) into separate Rag1−/− recipients. Animals that received Ndfip1-deficient cells lost much more of their initial body weight than those that received control cells (Fig. 3j). Furthermore, recipient animals that received T cells lacking Ndfip1 showed a higher inflammation index (Fig. 3k), increased lung cellularity, and had significantly higher percentages and numbers of Th17 cells in their lungs (Fig. 3l). These data are consistent with the hypothesis that Ndfip1 deficient Th17 cells accumulate and drive more pathology *in vivo*.

### Ndfip1 limits secretion of IL-17A upon restimulation of Th17 cells and mediates ubiquitination and degradation of RORγT

**Figure 4:**
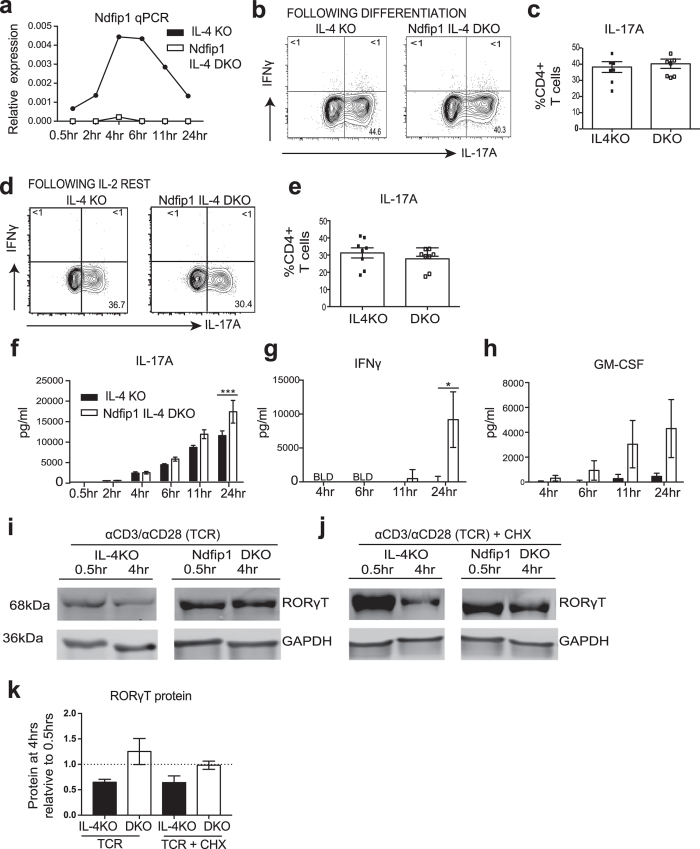
Ndfip1 limits proinflammatory cytokine production from Th17 cells upon restimulation and promotes RORγT degradation. (**a**) Ndfip1 mRNA expression, relative to beta actin, in restimulated Th17 cells over multiple time points indicated. (**b**) Representative plot showing IL-17A^+^ and IFNγ^+^ cells after the initial 5 days of Th17 polarization. (**c**) Summary data for (**b**) over multiple experiments. (**d**) Representative plot showing IL-17A^+^ or IFNγ^+^ Th17 cells after IL-2 expansion of cells shown in panels b and c. (**e**) Summary of data over multiple experiments. (**f**–**h**) Cytokines were analyzed by ELISA following restimulation of the Th17 cells. (**f**) IL-17A (**g**) IFNγ and (**h**) GM-CSF. (**i**-**k**) Naïve CD4+ T cells were differentiated into Th17 cells and then restimulated with αCD3/CD28 (TCR) for 0.5 hrs or for 4 hrs in the absence (**i**) or presence (**j**) of cycloheximide (CHX). Lysates were analyzed for RORγT and GAPDH levels using western blot. (**k**) Quantification over multiple experiments of RORγT levels relative to GAPDH loading control. In (**a**–**h**) n = 8 per genotype analyzed in 4 independent experiments. Panel e is a summary of cells used in (**f**–**h)**. Panels i-k represent data from n = 4 per genotype in 3 independent experiments. Significance was calculated by unpaired T tests for panels c and e and by 2-way ANOVA for panels f-h. *p < 0.05, ***p < 0.001. All error bars represent mean +/− SEM.

Given that Th17 cells lacking Ndfip1 were able to accumulate *in vivo*, and their heightened frequency correlated with increased pathology, we hypothesized that Ndfip1-deficient Th17 cells could be potent producers of proinflammatory cytokines. Limiting cytokine secretion from effector Th17 cells and controlling the formation of memory Th17 cells helps to ensure long-term defense against extracellular pathogens^38^,^39^. To begin to determine whether Ndifp1 regulates proinflammatory cytokine production in Th17 cells, we first tested when Ndfip1 was expressed in Th17 cells after TCR stimulation. We generated Th17 cells *in vitro*, expanded them in IL-2, and then restimulated them on plate-bound anti-CD3 and anti-CD28 for various time points, and analyzed Ndfip1 expression by qPCR. Cells lacking IL-4 were used since Ndfip1-deficient T cells secrete high levels of IL-4 which inhibit *in vitro* Th17 differentiation^27^. We found that Ndfip1 levels increased over the first 6 hours, and then returned close to base line levels by 24 hours (Fig. 4a). These data suggested that Ndfip1 might be particularly functional between 4 and 24 hours after restimulation. To prepare for testing Th17 producing cytokines, we first wanted to ensure that Ndfip1-deficient and control cells had similar numbers of Th17 cells following IL-2 expansion. Thus, we tested the cells directly following differentiation, and after expansion for percentages of cells expressing IL-17A and IFNγ. We found, as in prior experiments, that cells lacking Ndfip1 and control CD4 T cells were equally likely to differentiate into Th17 cells that expressed IL-17A but not IFNγ (Fig. 4b and c). As has been reported by several other groups^40^, we noticed a slight decrease in the percentage of IL-17A+ cells in culture after three days of IL-2 expansion (Fig. 4d and e). Nevertheless, the decrease in frequency of IL-17A+ cells was quite similar in both Ndfip1-deficient and Ndfip1-sufficient IL-4 KO cells T cells and thus an equal number of these cells were placed on an anti-CD3 and anti-CD28 -coated plate for restimulation. We then examined the secretion of IL-17A and other proinflammatory cytokines that can be made by Th17 cells. By 6 hrs post stimulation, Th17-polarized cells lacking Ndfip1 had already begun to secrete more IL-17A into culture, compared to their Ndfip1-sufficient counterparts (Fig. 4f) and by 24 hours the IL-17A in the Ndfip1-deficient Th17 culture supernatant was significantly higher than in cultures of control cells. Importantly, this time point correlated with the peak of Ndfip1 expression in control cells. Furthermore, when the supernatant was assayed to detect other Th17-related cytokines, we found that the Ndfip1 IL-4 DKO Th17s secreted more IFNγ (Fig. 4g) and GM-CSF (Fig. 4h) than their Ndfip1-expressing control counterpart cells. These data indicate that Ndfip1-deficient Th17 cells secrete more proinflammatory cytokines. Another interpretation of the data, could be that there were more IFNγ+ Th1 cells in the Ndfip1-deficient cultures prior to stimulation, however the plots in Fig. 4d do not support this conclusion and when the same experiment was performed using Th1 polarizing conditions, we found that Ndfip1-deficient Th1 cells secrete less IFNγ than controls upon restimulation (Supplementary Fig. S2). Thus, we conclude that Ndfip1 limits the secretion of proinflammatory cytokines from Th17 cells upon re-encounter with TCR stimulus.

RORγT is the lineage-defining transcription factor for Th17 cells^4^ and has been shown to drive the transcription of cytokines such as IL-17A^4^ and GM-CSF^19^,^20^. Since Ndfip1-deficient Th17 cells produce higher amounts of IL-17A and GM-CSF upon restimulation, we hypothesized that Ndfip1-deficient cells might fail to degrade RORγT. Supporting this, it was recently shown that Itch ubiquitylates and degrades RORγT^30^. Given that Ndfip1 can help to activate Itch in T cells^24^, we tested whether in the absence of Ndfip1, RORγT fails to be degraded. We generated Th17 cells from Ndfip1 IL-4 DKO, and IL-4 KO control cells. We then restimulated these cells for 0.5 hours or 4 hours (Fig. 4i) and analyzed lysates for levels of RORγT. We found that Ndfip1-sufficient Th17 cells decreased their levels of RORγT between 0.5 hours and 4 hours. In contrast, Th17 cells lacking Ndfip1 maintained RORγT protein levels after stimulation. To investigate whether the decrease seen in Ndfip1-sufficient Th17 cells was due to RORγT protein degradation, we stimulated the Th17 cells in the presence of cycloheximide (Fig. 4j). Indeed, IL-4KO Th17 cells showed a significant loss of RORγT protein levels after 4 hours of stimulation in the presence of cycloheximide, in contrast, Ndfip1 IL-4 DKO Th17 cells maintained their RORγT levels (Fig. 4k). Taken together, these data support that Ndfip1 is important for normal RORγT protein degradation after TCR stimulation.

### Itch E3 ligase is required for restricting Th17 cell abundance *in vivo*

**Figure 5:**
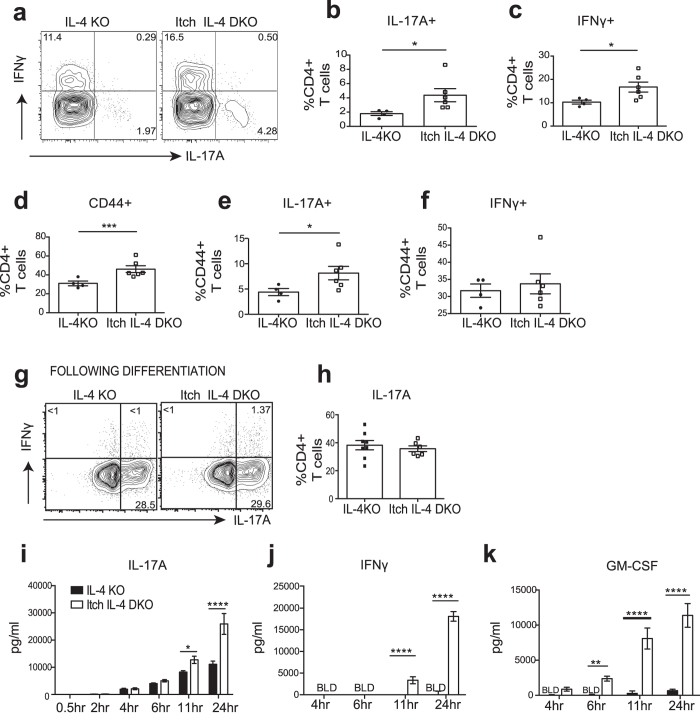
Itch E3 ligase is required to restrict numbers of Th17 cells and secretion of proinflammatory Th17 cytokines. (**a**–**f**) Flow cytometric analysis of lung cells from Itch IL-4 DKO mice or IL-4KO controls. (**a**) Percentage of IL-17A^+^ or IFNγ^+^ CD4^+^ cells. (**b**,**c**) Percentage of IL-17A^+^ (**b**) or IFNγ^+^ (**c**) among CD3^+^ CD4^+^ lung cells. (**d**–**f**) Percentages of previously activated CD44^+^ lung cells among CD4+ T cells (**d**) and percentages of IL-17A^+^ (**e**) or IFNγ^+^ (**f**) previously activated cells shown in panel D. (**g**–**k**) Naïve sorted CD4+ T cells were differentiated into Th17 cells for 5 days and expanded in IL-2. (**g**) Representative plot showing IL-17A^+^ or IFNγ^+^ Th17 cells after differentiation. (**h**) Summary of data over multiple experiments. (**I**,**k**) Cytokines were analyzed using ELISA following restimulation of equal numbers of IL-2 expanded Th17 cells. (**i**) IL-17A (**j**) IFNγ and (**k**) GM-CSF. In all summarized plots, each dot represents an individual mouse analyzed in at least 2 independent experiments. Significance was calculated by unpaired T tests for panel b–f, h and by 2-way ANOVA for panels i-k. *p < 0.05, **p < 0.01,***p < 0.001, ****p < 0.0001. All error bars represent mean +/− SEM.

Ndfip1 is a well-known activator of the E3 ligase, Itch and related Nedd-4 family E3 ubiquitin ligases. Loss of Itch results in a spontaneous, auto-inflammatory, Th2-predominant disease, similar to that seen in Ndfip1-deficient animals^41^, albeit with a delayed onset. Recently it was shown that mice lacking Itch have hyper-functional Th17 cells^30^. To investigate whether Itch, like Ndfip1, is required for the regulation of Th17 cell numbers and cytokine production, we generated Itch IL-4 DKO animals and examined Th17 cells in their lungs. We found that, similar to mice lacking both Ndfip1 and IL-4, the absence of Itch and IL-4 led to an increase in Th17 cells and IFNγ+ cells (Fig. 5a–c). Furthermore, Itch IL-4 DKO CD4+ T cells were more likely to be activated (Fig. 5d) and while this increase in activated cells could explain the increase in IFNγ+ CD4+ T cells (Fig. 5f), it failed to explain the increase in Th17 cells in these animals (Fig. 5e). Additionally, as was seen with the CD4+ T cells lacking Ndfip1, there was no increased propensity towards *in vitro* Th17 differentiation in Itch-deficient CD4 T cells (Fig. 5g and h). However upon re-stimulation, Itch-deficient Th17 cells were more likely to secrete significantly higher amounts of IL-17A, IFNγ, and GM-CSF (Fig. 5i–k). These data support that Ndfip1 and Itch limit Th17 cell numbers and proinflammatory cytokine secretion and this is distinct from the established roles for Ndfip1 and Itch in limiting IL-4 production from T cells and in limiting T cell activation.

### Ndfip1-deficient Th17 cells recruit more neutrophils and drive severe mucosal barrier destruction

Secretion of proinflammatory cytokines from Th17 cells is one way Th17 cells exert pathogenic functions *in vivo*. These cytokines act on non-hematopoietic cells such as stromal cells, leading to their secretion of proteins, including chemokines, which recruit other immune cells to sites of inflammation. Additionally, Th17 cell cytokines can directly recruit neutrophils. Uncontrolled recruitment of immune cells to the site of inflammation increases the likelihood of immunopathology. Mice that constitutively lack Ndfip1 develop inflammation in the small bowel and colon by 6 weeks of age, characterized by increased frequencies of eosinophils, likely as a consequence of Th2-inflammation^34^, and neutrophils. This inflammation was not seen when these mice are bred onto a RAG−/− background and therefore lack B and T cells^34^. We examined the colons in mice lacking Ndfip1 only in their T cells (cKO) or control animals at 10 weeks of age. We observed spontaneous disruption of the mucosal layer of the colon epithelium in the cKO animals (Supplementary Fig. S3). Furthermore, colons from cKO mice showed higher frequencies of neutrophils (Supplementary Fig. S3). However, since the cKO mice are potent IL-4 producers, and IL-4 mediated inflammation can also lead to colon inflammation, it was not clear whether the increased neutrophils in the colons was driven by an increase in Th17 cells in these mice.

**Figure 6:**
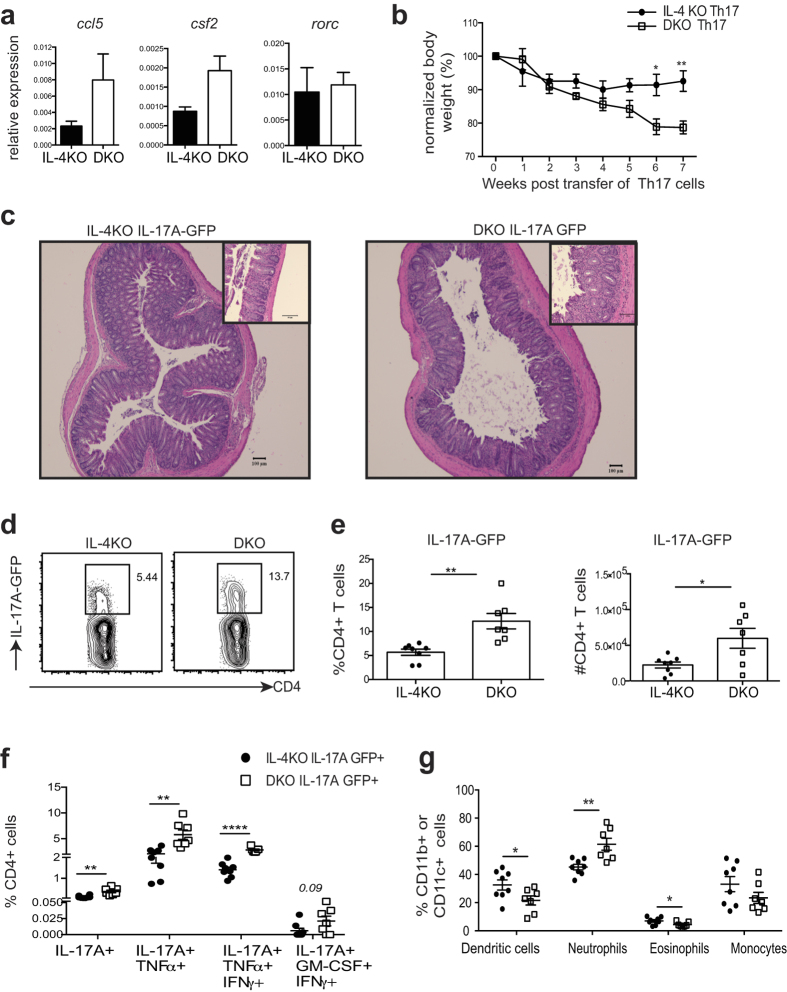
Th17 cells lacking Ndfip1 are more likely to produce proinflammatory cytokines and drive tissue destruction. CD4^+^ naïve cells from IL-4 KO or DKO T cells from IL-17A GFP reporter mice were differentiated into Th17 cells for 5 days. GFP-IL-17A^+^ Th17 cells from these cultures were sorted and analyzed for gene expression via qPCR. (**a**) mRNA expression for *ccl5* (left panel), *csf2* (middle panel) *rorc* (right panel) was analyzed and is shown relative to beta-actin. (**b**–**g**) These same cultures were sorted for Ndfip1-deficient and -sufficient GFP-IL-17A^+^ Th17 cells that were then injected into RAG1^−/−^ recipients. Mice were analyzed for weight loss (expressed as percentage of starting weight) (**b**) and colon integrity (**c**) using Hemaoxylin and Eosin (H&E) staining. Inset represents a separate higher magnification image of the colon. All size bars represent 100 um. (**d**,**e**) Percentages and absolute numbers of Th17 cells in the colons as determined by flow cytometry. (**f**) Boolean analysis of flow cytometry data from DKO versus IL-4KO colon CD3^+^ CD4^+^ cells that secrete IL-17 or IL-17 together with other cytokines. (**g**) Frequencies of CD11b+ Ly6G+ neutrophils, CD11c+ MHCII+ dendritic cells, CD11b+ Siglec F eosinophils, and CD11b+ Ly6G- Ly6C+ monocytes in the colon as determined by flow cytometry. Panel a, n = 3 IL-4KO and n = 4 DKO GFP-IL-17A^+^ Th17 cells collected from 3 independent experiments. For panels b–g, data is pooled for n = 8 recipients of each genotype in two independent experiments. *p < 0.05, **p < 0.01,****p < 0.0001. p values were calculated by unpaired two-tailed T tests. All error bars represent mean +/− SEM.

Thus we sought to test whether the increased production of proinflammatory cytokines by Th17 cells lacking Ndfip1 would drive increased pathology in an environment free of IL-4, in order to focus only on the role of Ndfip1 in Th17 cells and to exclude IL-4-mediated effects. To do this, we generated Ndfip1−/− IL-4−/− IL-17A-GFP mice, and IL-4−/− IL-17A-GFP controls, that lack both Ndfip1 and IL-4 and that harbor a fluorescent GFP-reporter that identifies, and thus allows purification of, Th17 cells. We then differentiated CD4+ T cells under Th17 polarizing conditions, as detailed above, sorted IL-17A-GFP+ (Th17) cells, and injected them into RAG1−/− recipients or analyzed them by qPCR for mRNA expression. We found that Ndfip1-deficient Th17 cells showed higher expression of *ccl5* and *csf2* (which encodes GM-CSF) (Fig. 6a, left and middle panels), factors associated with Th17 cell pathogenicity^18^. We also analyzed *rorc* (which encodes RORγT), which was not different between Ndfip1-sufficient and -deficient Th17 cells (Fig. 6a, right panel), supporting that increased RORγT in Ndfip1-deficient cells is not due to increased transcription. If expanded in IL-2, Ndfip1-deficient Th17 cells similarly showed higher expression of *csf2* and *ccl5* (Supplementary Fig. S4) and furthermore, showed higher expression of the Th17-related cytokines *ifng* and *tnfa* but not *il21* (Supplementary Fig. S4) compared to Ndfip1-sufficient Th17 cells. The gene expression levels for transcription factors such as *rorc, tbx21* (which encodes T-bet) and *blimp1*, which have all been associated with Th17 cell pathogenicity were not different between Ndfip1-sufficient and -deficient Th17 cells (Supplementary Fig. S4).

We then assessed the function of these cells *in vivo*. By approximately 6 weeks after transfer of purified Th17 cells, mice that received Ndfip1-deficient Th17 cells had lost significantly more body weight than their Ndfip1-sufficient controls (Fig. 6b). Additionally, colon histology revealed that recipients of Ndfip1-deficient Th17 cells had higher incidence of transmural inflammation and erosion of normal mucosal structure (Fig. 6c). Strikingly, in all recipient mice, less than 20% of all CD4 T cells recovered from the colons, whether expressing or lacking Ndfip1, were still IL-17A+, indicating the remarkable instability of the Th17 cells in this model (Fig. 6d and e). However, mice which received Ndfip1-deficient Th17 cells had a higher frequency of IL-17A+ cells in the colon, suggesting that Ndfip1 helps to limit Th17 stability (Fig. 6d and e). Considering our prior results, we also tested other cytokines produced by Th17 cells including: TNFα, IFNγ, GM-CSF and IL-10. We found that Ndfip1-deficient CD4 cells in the colon were not only more likely to be making exclusively IL-17A, but we also found more Ndfip1-deficient IL-17A+ cells that were simultaneously making TNFα, TNFα with IFNγ, or GM-CSF with IFNγ (Fig. 6f). Given the well-described role of TNFα and IL-17A in driving neutrophil recruitment to sites of inflammation^42^,^43^,^44^,^45^,^46^,^47^, we examined the numbers of neutrophils in the colons of the mice that received Ndfip1-deficient or –sufficient Th17 cells. We found that recipients of Ndfip1-deficient Th17 cells had significantly higher numbers of neutrophils (Fig. 6g). This was not true for other innate cells examined such as eosinophils, dendritic cells, or monocytes (Fig. 6g). Thus, Ndfip1 limits production of proinflammatory cytokines such as IL-17A, IFNγ, and TNFα; Th17 stability; and Th17-mediated pathology.

## Discussion

Ndfip1 and Itch are known to limit the activation of T cells, as well as to control the differentiation of specific lineages of T cells, namely Th2 cells and peripherally-generated Foxp3+ regulatory T cells^24^,^29^,^41^. Less is known about whether and how these proteins impact the fate or function of T cells that have already committed to a cytokine-producing effector cell lineage. Here, we show that these two proteins play an important role in Th17 cells after differentiation. Specifically, our data reveal a new role for these proteins in limiting the abundance and pathogenicity of Th17 cells and show that Ndfip1 and Itch regulate the effector responses of Th17 cells following lineage specification.

This study focused predominantly on Ndfip1, an activator of Itch catalytic activity^24^,^28^,^48^,^49^. It has been shown that the loss of Ndfip1 globally, or following conditional deletion in T cells, causes spontaneous autoinflammatory disease that is associated with high numbers of activated CD4 T cells, increased numbers of Th1, Th2, and Th17 cells, and premature death^24^,^26^,^27^,^29^. Prior studies have detailed how Ndfip1 limits Th2 cell numbers and how the production of IL-4 can drive much of the pathological sequela^27^,^29^. In this study, we made use of Ndfip1 IL-4 DKO animals to show that while IL-4 over-production by Ndfip1-deficient T cells may influence the generation of Th17 cells *in vivo* according to previous reports^27^, Ndfip1 has a cell-intrinsic role in the regulation of already-differentiated Th17 cells. This study also reveals, for the first time, that Ndfip1 limits the numbers of Th1 and Th17 cells by two distinct mechanisms. We show that Ndfip1 restricts T cell activation to limit the numbers of Th1 cells. In contrast, Ndfip1 limits Th17 cell abundance by dampening Th17 cell proliferation and by working together with Itch to degrade RORγT.

In Th17 cells lacking Ndfip1, RORγT levels accumulate and the production of proinflammatory cytokines and chemokines is increased. Some of these factors are known to be transcriptional targets of RORγT, namely GM-CSF, CCL5 (a.k.a. RANTES) and IL-17A, while others, IFNγ and TNFα, are not^4^,^19^,^50^. Thus, it remains unclear whether the increase in RORγT is sufficient to explain the full pathogenic potential of Th17 cells that lack Ndfip1, or whether there remain other substrates of Ndfip1/Itch yet to be identified. We propose that Ndfip1 helps to degrade RORγT and thus limits the production of CCL5, GM-CSF and IL-17A by Th17 cells.

IFNγ production by Th17 cells is mechanistically distinct from its production by Th1 cells. In Th17 cells, IFNγ synthesis does not depend on Tbet, STAT1 or STAT4^51^, and instead is linked to IL-23R signaling and the transcription factor Blimp1^51^. Ndfip1-deficient Th17 cells do not show increased levels of IL-23R mRNA compared to WT cells, however we have not explored IL23R protein levels or protein levels of Blimp1. Blimp1 can also increase the production of IFNγ, GM-CSF, and IL-17A from Th17 cells^8^,^17^. Interestingly, Blimp1, like RORγT, contains an LPXY motif that would allow binding to Itch. Thus, future studies should determine whether, like RORγT, Blimp1 is a substrate of Itch/Ndfip1.

Ndfip1-deficient Th17 cells concurrently produce TNFα, IFNγ, and IL-17A *in vivo*. TNFα, also called cachexin, is an important mediator of systemic inflammation. Mechanistically, TNFα may suppress appetite, or may act synergistically with IFNγ to target the degradation of myosin heavy chains leading to muscle wasting^52^. Thus concurrent production of TNFα and IFNγ by Ndfip1-deficient Th17 cells, coupled with the observed destruction of the colon mucosa, may explain the severe weight loss seen in the colitis model. Like IFNγ, TNFα production is unlikely to be driven by RORγT since TNFα expression in Th17 cells increases upon loss of RORγT^50^. How Ndfip1 regulates TNFα production is unknown and remains to be explored.

Recently published work found that Itch ubiquitylates RORγT, targeting it for degradation and helping to limit the abundance of Th17 cells in the colon^30^. We found that Ndfip1 also controls RORγT levels. Furthermore, our data revealed that Ndfip1 and Itch do not merely limit Th17 abundance at mucosal sites, but also limit the pathogenic potential of these cells by restricting the amount of RORγT and, consequently, the production of proinflammatory cytokines that Th17 cells secrete. We find that in the absence of Ndfip1 or Itch, Th17 cells secrete increased amounts of IL-17A, IFNγ, GM-CSF and TNFα. Of these cytokines, increased amounts of IL-17A and TNFα are known to promote colorectal cancer while the role of GM-CSF in colorectal cancer remains unclear^53^,^54^. Our data suggests that the observed remarkable susceptibility of Itch-deficient mice to colitis and colorectal cancer^30^ is not only because of the quantity of Th17 cells found in the colon, but also because of the pathogenic potential of these Th17 cells.

We found that the loss of Ndfip1 or Itch does not lead to increased differentiation of Th17 cells. Thus, Ndfip1 and Itch are primarily regulating RORγT levels after the cells have increased RORγT and committed to the Th17 lineage. Interestingly, while both Ndfip1 and Itch promote RORγT degradation, Th17 cells lacking Itch produce higher levels of IL-17A, IFNγ and GM-CSF than Ndfip1-deficient cells. This suggests that while Itch may require Ndfip1 for its activation in Th17 cells, it may also rely on other mechanisms of activation. We recently found that Ndfip2 has similar functions to Ndfip1 and can also activate Itch and related E3 ubiquitin ligases in CD4 T cells^28^. It remains to be seen whether Ndfip2 may also work with Itch to limit Th17 cell proinflammatory cytokine production.

This work may have relevance in the development or treatment of diseases such as asthma. A sub-set of patients with asthma do not respond to steroids or other standard treatments and these patients are much more likely to have neutrophilic accumulation in the airways^55^,^56^. Emerging data suggests that neutralization of either Th2 cytokines alone or of Th17 cytokines alone in asthma may worsen disease, resulting in increased inflammation in the airways^57^. However, neutralization of both Th2 and Th17 cytokines ameliorate neutrophilia, eosinophilia, and overall airway inflammation^57^. Our data reveals Ndfip1 as a key regulator of both Th2- and Th17-cell functions, albeit via separate mechanisms. Therapeutics designed to mimic Ndfip1, and activate Itch, would be predicted to have high efficacy in treating asthma while having a minimal effect on anti-viral immune responses. Such therapies will be the focus of future studies.

## Materials and Methods

### Mice

Ndfip1^−/−^, Ndfip1^fl/fl^ CD4-Cre^+^, IL-4^−/−^ and Ndfip1^−/−^ IL-4^−/−^ (DKO) mice have been described previously^24^,^25^,^27^,^34^. IL-17A GFP mice were purchased from Jackson laboratory [stock 018472], were crossed to in-house IL-4^−/−^ or Ndfip1^−/−^ IL-4^−/−^ (DKO) mice, and bred for one copy of the IL-17A GFP gene. Itch IL-4 DKO mice were generated by crossing previously described Itch^−/−^ animals^41^ to in-house IL-4^−/−^ animals. CD45.1 IL-4^−/−^ mice were obtained by crossing in-house CD45.1 (C57BL6.SJL-Ptprc^a^ Pepc^b^/BoyJ) mice to in-house IL-4^−/−^ animals for more than 10 generations. The T cell transgenic B3K506 mice have been described previously^36^,^58^, were obtained from the laboratory of Eric Huseby, and crossed to Ndfip1^−/−^ Rag1^−/−^ animals. In all experiments described, male and female mice were appropriately age and sex-matched in experiments and data was pooled.

All mice were maintained in a barrier facility at the Children’s Hospital of Philadelphia and experiments were approved and in accordance with guidelines established by the Children’s Hospital of Philadelphia Institutional Animal Care and Use Committee. Primers for Ndfip1^−/−^, CD4-Cre^+^, IL-4^−/−,^ IL-17A GFP, Rag1^−/−^ mice are available on the Jackson Laboratories website (https://www.jax.org/). CD45.1 mice were genotyped by staining peripheral blood lymphocytes for CD45.1 versus CD45.2, while B3K mice were genotyped by staining peripheral blood lymphocytes for B220^-^, CD4^+^ Vb8.1^+^ T cells.

### Mixed chimeras

Fetal liver was harvested from embryos between days E13-E17 old. Fetal livers were crushed through a 35 um FACS tube with strainer cap and washed with plain DMEM media. After centrifugation, pellets were resuspended in 90% FCS 10% DMSO freezing media and stored at −80 °C until needed. Bone marrow was harvested from donor mice by flushing the femurs and tibias with a 22G needle and 10 ml syringe containing DMEM. The flushed cells were strained though a 70 um strainer cap and 5 ul of purified anti CD4 (clone GK1.5) and anti CD8 (clone 53-6.7) antibodies were added to 5 ml of bone marrow cells and rotated at 4 degrees for 30 mins-1 hr. Cells were washed with extra DMEM and resuspended in 1 ml of suspension. 1.5 ml of goat anti rat IgG magnetic beads were washed in a dynamagnet and the 1 ml of bone marrow suspension was added to the washed beads and placed on a dynamagnet. Supernatant containing untouched, T cell depleted bone marrow was collected, washed, resuspended in freezing media and frozen at −80 °C until needed. RAG1^−/−^ recipient animals were sublethally irradiated with 400 cGy 18–24 hours before injection. Frozen fetal liver or T cell-depleted bone marrow was thawed and resuspended in sterile PBS. The irradiated mice were reconstituted with a total of 0.5–1.0 × 10^6^ total cells per 100 ul via the intravenous route (tail vein or retroorbitally). Mice were allowed 7–10 weeks for reconstitution. No difference was observed between fetal liver versus bone marrow-reconstituted animals.

### Histological analysis

Stool was removed from colons by lightly pressing out using curved forceps. 1–2 mm sections of the most distal colon were taken and fixed in 10% neutral buffered formalin for at least 24 h. Tissues were then paraffin-embedded and sectioned to 5 μm thickness and stained with Hematoxylin and Eosin (H&E). Images were obtained using a Leica DM4000B upright scope paired with a Spot RT/SE Slider camera with 5x, 10x, and 20x objective lens.

### Tissue processing for flow cytometry

After transcardial perfusion of lungs and flushing colons with cold HBSS buffer, they were minced with DMEM (Dulbecco’s minimal essential medium) containing 0.9 mg/ml of collagenase A, 0.8 mg/ml of collagenase 1 A, and 20 μg/ml of DNase I and rotated end-over-end for 1 h at room temperature. FCS was added at 15% v/v and the mixture was filtered sequentially through a 100 um and 40 um filter before centrifugation. Lung pellets were RBC-lysed using in-house ammonium-chloride-potassium (ACK) lysis buffer, washed, resuspended in complete DMEM media and refiltered. Spleens and thymi were crushed with the back end of a 1 ml syringe through a 70 um filter and into a petri dish containing complete DMEM media. Thymi were directly resuspended in complete media for staining while spleens were RBC-lysed before being resuspended and filtered in complete T cell media for flow cytometry. Complete T cell media contained DMEM, 10% FCS, 1% Penicillin/Streptomycin, 1% glutamax and 0.12 mM beta-mercaptoethanol. Tissue homogenates were stimulated with PMA (30 ng/ml), Ionomycin (1 uM) and Golgi plug inhibitor (1:1000, BD Biosciences) for 4 hrs at 37 degrees in a 5% CO_2_ incubator. Surface and intracellular staining were performed for surface antigens and cytokines, respectively, before flow cytometric analysis. For flow cytometry panels where only surface proteins were required, no PMA/Ionomycin stimulation was performed.

### Flow cytometry

Freshly prepared or PMA/Ionomycin-stimulated tissue homogenates in FACs tubes were washed once with PBS and stained at 1:60 with 10 ul live dead blue viability stain for 5 mins on ice, stained for an additional 5 mins with 50 ul Fc block at 1:25 (anti-CD16/32, clone 2.4G2) and then for 20 mins on ice with 50 ul of cell surface stains. Cells were then fixed overnight (~16 hours) with the foxp3 ebioscience fix/perm kit and stained for intracellular proteins for 45 mins on ice. Flow cytometry data were acquired on an LSR Fortessa and analyzed using Flowjo software (Tree Star). Events analyzed were: dye-negative cells; FSC-A by FSC-H singlet cells; SSC-H by SSC-W singlet cells; and FSC A by SSC A lymphocytes. The following antibodies were used for flow cytometry: anti CD4 (ebioscience, GK1.5); anti IL-17A (ebioscience, eBio17B7,), anti CD3 (Biolegend, 17A2); anti-CD44 (Biolegend, IM7); anti TNFα (BD, MP6-XT22), anti CD25 (ebioscience, PC61.5); anti CD45.2 (Biolegend, 104); anti CD45.1 (Biolegend, A20), anti Ly6G (BD, 1A8), anti Ly6C (Biolegend, HK1.4); anti Ki67 (Biolegend, 16A8); anti GM-CSF (Biolegend, MP1-22E9); anti RORγT (ebioscience, B2D); anti Siglec F (BD, E502440); anti CD11b (Biolegend, M1/70) and anti IL-10 (ebioscience, JES5-16E3).

### *In vitro* T cell differentiation protocols

Single cell suspensions of spleen and lymph nodes were sorted on a Moflo Astrios for naïve CD4+ cells (CD8− CD44− CD62L+ CD25−). 0.5 × 10^6^ naïve cells were cultured for 5 days in Th17 polarizing media containing TGF-β (PeproTech), 20 ng/ml IL-6 (R&D Systems), 20 ng/ml IL-1β (PeproTech) and 50 ng/ml IL-23 (R&D Systems). In the experiments shown in Fig. 2a and b, 5 ng/ml of TGFβ was used; all other experiments used 0.5 ng/ml. For Th1 polarizing conditions, 50 U/ml (~10 ng/ml) rhIL-2 and 20 ng/ml IL-12 were added to the media. In both cases, cells were cultured on 24 well plates coated with 500 ul of 5 ug/ml anti-CD3 (clone 145-2C11) and 5 ug/ml anti-CD28 (clone 37.51) antibodies. After harvest, cells were restimulated with PMA (30 ng/ml), Ionomycin (1 uM) and Golgi plug inhibitor (1:1000) for 4 h before intracellular cytokine staining.

### Th17 differentiation of B3K Trangenic (Tg) T cells

CD4+ T cells were isolated from 7–12 week old Rag1^−/−^ B3K Tg or Ndfip1^−/−^ Rag1^−/−^ B3K Tg animals and incubated for 5 days in Th17-polarizing media: and in the presence of 20 ug/ml anti-IL-4. Each well also contained 10 × 10^5^ congenically marked (CD45.1) APCs pulsed for 2 hours with 10 ug/ml of peptide (p3K, p-1K or p-1A). On day 5, cells were harvested and restimulated with PMA (30 ng/ml), Ionomycin (1 uM) and Golgi plug inhibitor (1:1000) for 4 hrs before intracellular cytokine staining.

### ELISA

5 days after differentiation, Th1 or Th17 cells were harvested, re-plated at 1 × 10^6^/ml in 50 U/ml IL-2 and expanded for 72 h, splitting cells daily. After 3 days, cells were resuspended at 1 × 10^6^ cells/ml and 0.5 × 10^6^ of these Th1 or Th17 cells were restimulated via plate-bound 5 ug/ml anti-CD3/CD28 for 0.5, 2, 4, 6, 11 or 24 hrs in 48 well plates. Supernatants were collected and kept frozen at −80 °C until needed. ELISAs were carried in out triplicate and according to the manufacturer’s protocols for IL-17A (ebioscience, 88-7371), IFNγ (ebioscience, 88-7314) and GM-CSF (ebioscience, 88-7334). ELISA plates were read using a Synergy HT Microplate Reader.

### qPCR

Cultured cells were harvested with sterile PBS, resuspended in trizol reagent and frozen at −80 °C until needed. RNA was extracted using the RNeasy Mini Kit (Qiagen). Isolated RNA was converted to cDNA using the High Capacity RNA-to-cDNA kit (Applied Biosystems, #4387406). qPCR plates were set up using the Taqman Gene expression master mix (Applied Biosystems, #4369514) and primers for Ndfip1^29^, Actin (4352933E), ccl5 (Mm01302427_m1), csf2 (Mm01290062_m1), rorc (Mm01261022_m1), tbx21 (Mm00450960_m1), tnf (Mm00443258_m1), ifng (Mm01168134_m1), blimp1/prdm1(Mm00476128_m1), all from Applied Biosystems. Each qPCR sample was run in triplicate on a 7500 Real-Time PCR system (Applied Biosytems) using 8–10 ng/ul of cDNA. Relative mRNA expression was calculated as 2^d^C_t_, where dC_t_ represents threshold cycle (C_t_) of actin beta minus C_t_ of gene of interest.

### Western blotting

Naïve CD4+ T cells were differentiated into Th17 cells for 5 days using 0.5 ng/ml TGFβ, 20 ng/ml IL-6, 20 ng/ml IL-1β, 50 ng/ml IL-23 and plate bound 5 ug/ml αCD3/CD28. On day 5, Th17 cells were harvested and resuspended at 1–2 × 10^6^ cells/ml and restimulated with plate bound 5 ug/ml αCD3/CD28 for 0.5 hrs or 4 hrs +/− 10 ug/ml final concentration of cycloheximide. Harvested cells were washed with PBS and lysed by incubating pellets with 1% Nonidet P-40 lysis buffer on ice for 20 mins, followed by spinning at 4 degrees at 15 000 rpm for 10 mins and saving the lysate. The lysis buffer contained 1% Nonidet P-40, 50 mM Tris-HCl (pH 8), 150 mM NaCl, 5 mM NaF, 1 mM Na Orthovanadate and complete protease inhibitors (Roche). 4x SDS sample buffer was added to lysate to a 1x dilution and this lysate was boiled for 10 mins at 95 degrees prior to loading on an SDS PAGE gel (Criterion pre-cast gels, Biorad). After transfer to a PVDF membrane, membranes were blocked for 1 hour with odyssey blocking buffer and probed with anti-RORγT and anti-GAPDH antibodies. RORγ (H-190) rabbit polyclonal from Santa Cruz was used at 1:1000 and GAPDH (mab374) mouse monoclonal from Millipore was used at 1:5000. Membranes were probed using anti-rabbit or anti-mouse IR800 and A680 secondary antibodies and imaged on an odyssey imager. Data was quantified using the Image Studio Lite software.

### Adoptive Th17 transfer experiments

WT or Ndfip1-deficient naïve CD4+ T cells were sorted from IL-17A GFP reporter mice (Ndfip1^−/−^ IL-4^−/−^ IL-17A-GFP versus IL-4^−/−^ IL-17A GFP) and differentiated into Th17 cells using 0.5 ng/ml TGFβ, 20 ng/ml IL-6, 20 ng/ml IL-1β, 50 ng/ml IL-23, 20 ug/ml anti-IL-2, and plate bound 5 ug/ml antiCD3/antiCD28 for 4–5 days. Pure Th17 cells were sorted as CD4+ (IL-17A)-GFP+ cells and 2 × 10^5^ of these cells were introduced via intraperitoneal injection (IP) into each recipient RAG^−/−^ host. After 5–7 weeks, animals were analyzed in experiments.

### Statistical analysis

All statistical analyses were performed using Graphpad prism software with p values less than or equal to 0.05 considered statistically significant. In mixed chimera experiments and in co-transfer experiments, p values were calculated via a paired t test. In *ex vivo* flow cytometric analysis, p values were calculated by unpaired test or ANOVA, as appropriate. In time course ELISA experiments, p values were calculated by 2-way ANOVA.

## Additional Information

**How to cite this article**: Layman, A. A. K. *et al*. Ndfip1 restricts Th17 cell potency by limiting lineage stability and proinflammatory cytokine production. *Sci. Rep.* **7**, 39649; doi: 10.1038/srep39649 (2017).

**Publisher's note:** Springer Nature remains neutral with regard to jurisdictional claims in published maps and institutional affiliations.

## Supplementary Material

Supplementary Figures
