# Supplementary material for: Ndfip1 restricts Th17 cell potency by limiting lineage stability and proinflammatory cytokine production
Source: Sci Rep. 2017 Jan 4;7:39649. doi: 10.1038/srep39649 (PMC5209687; doi:10.1038/srep39649)
Supplement: Supplementary Figures [file srep39649-s1.pdf]

Supplementary figures

Ndfip1 restricts Th17 cell potency by limiting lineage stability and proinflammatory cytokine production.

Awo Akosua Kesewa Layman[1], Stephanie Sprout[2], Dylan Phillips[2] and Paula M. Oliver [2,3,*]

[1]Medical Scientist Training Program, Perelman School of Medicine and Biomedical Graduate Studies at the University of Pennsylvania, [2]The Children's Hospital of Philadelphia, Cell Pathology Division, [3]Department of Pathology and Laboratory Medicine, Perelman School of Medicine, University of Pennsylvania.

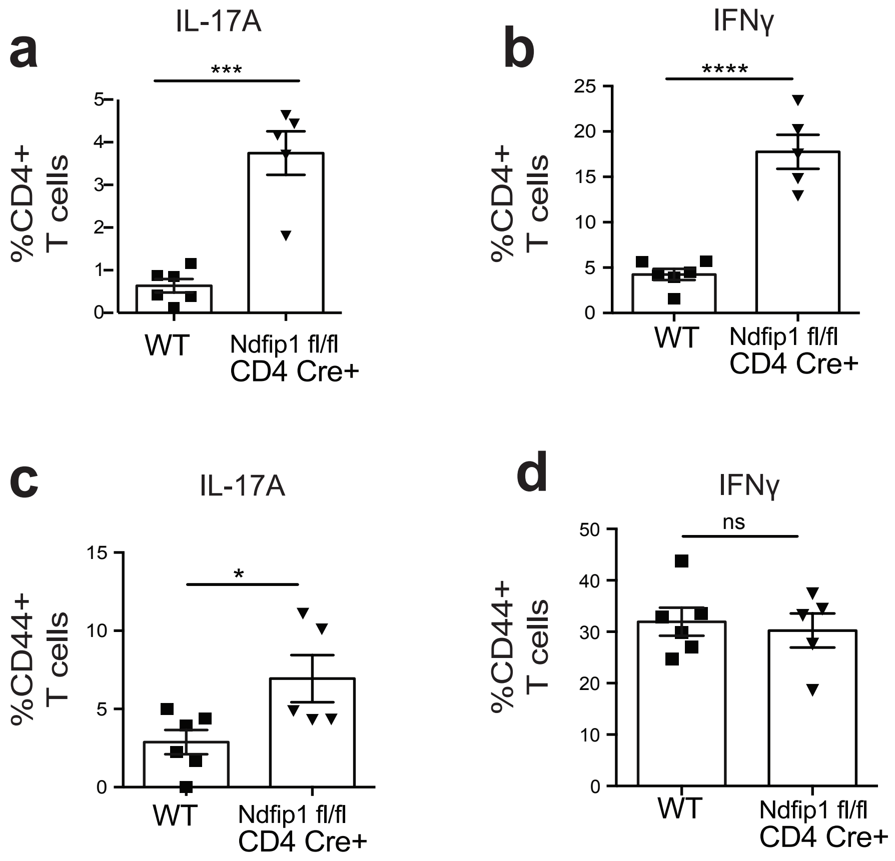

a
IL-17A
***
%CD4+ T cells
0
1
2
3
4
5
WT
Ndfip1 fl/fl CD4 Cre+
b
IFNγ
****
%CD4+ T cells
0
5
10
15
20
25
WT
Ndfip1 fl/fl CD4 Cre+
c
IL-17A
*
%CD44+ T cells
0
5
10
15
WT
Ndfip1 fl/fl CD4 Cre+
d
IFNγ
ns
%CD44+ T cells
0
10
20
30
40
50
WT
Ndfip1 fl/fl CD4 Cre+

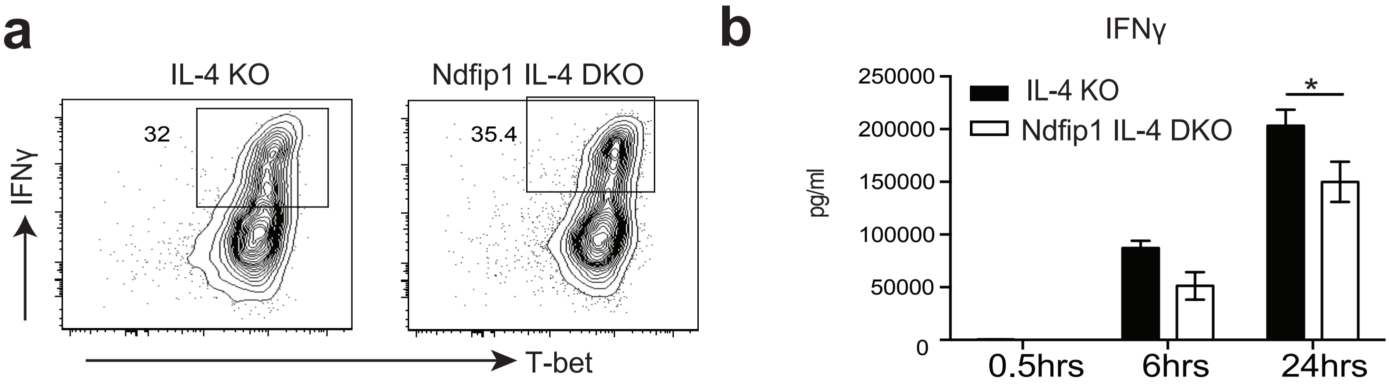

a
IL-4 KO
Ndfip1 IL-4 DKO
32
35.4
IFNγ
T-bet
b
IFNγ
IL-4 KO
Ndfip1 IL-4 DKO
*
pg/ml
250000
200000
150000
100000
50000
0
0.5hrs
6hrs
24hrs

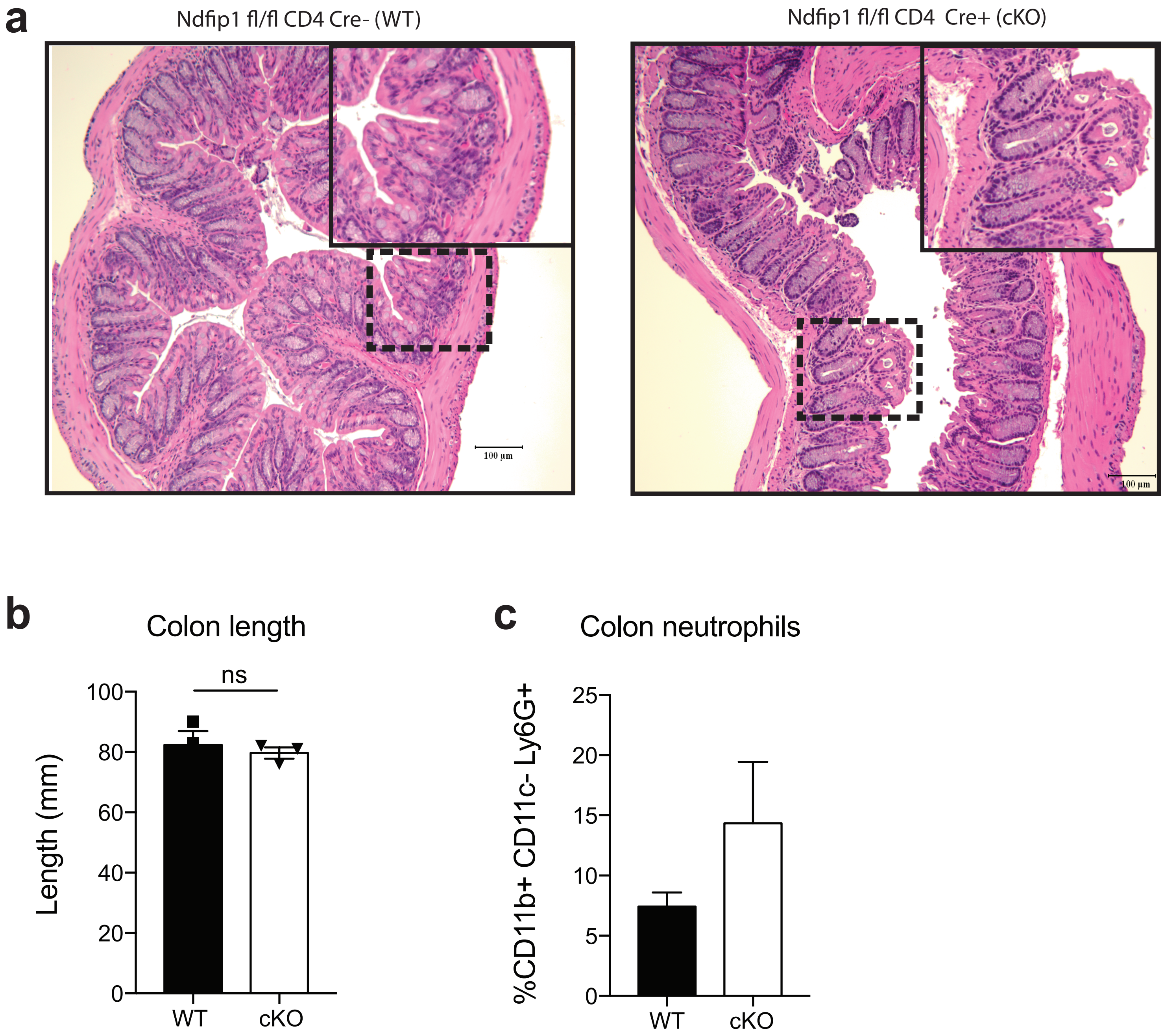
a
Ndfip1 fl/fl CD4 Cre- (WT)
Ndfip1 fl/fl CD4 Cre+ (cKO)
100 µm
100 µm
b
Colon length
ns
Length (mm)
100
80
60
40
20
0
WT
cKO
c
Colon neutrophils
%CD11b+ CD11c- Ly6G+
25
20
15
10
5
0
WT
cKO

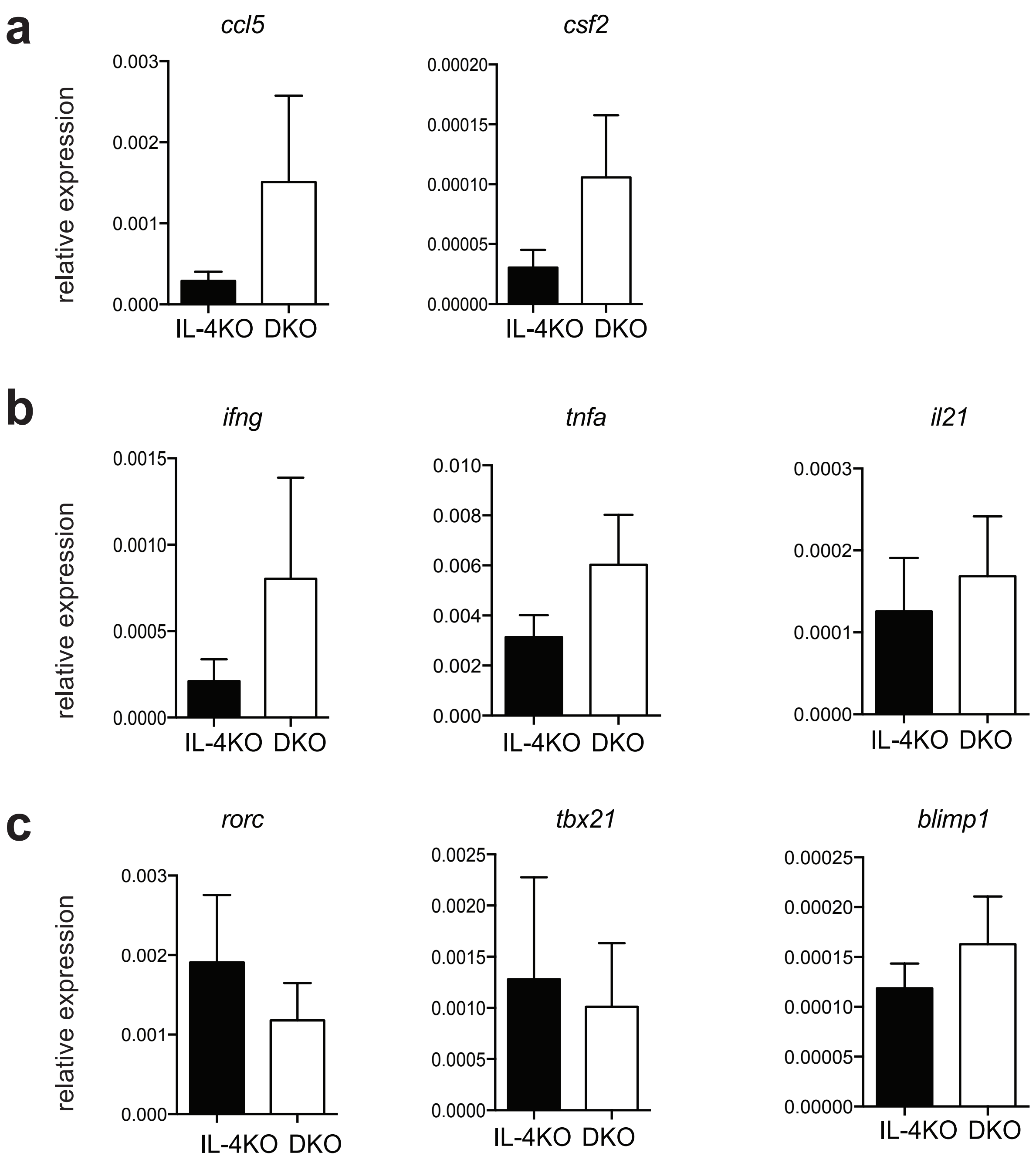

a
ccl5
csf2
b
ifng
tnfa
il21
c
rorc
tbx21
blimp1
relative expression
IL-4KO
DKO
0.003
0.002
0.001
0.000
0.00020
0.00015
0.00010
0.00005
0.00000
0.0015
0.0010
0.0005
0.0000
0.010
0.008
0.006
0.004
0.002
0.0003
0.0002
0.0001
0.0025
0.0020
0.00025

Supplementary Figure S1. Ndfip1 limits the abundance of Th17 cells.
Flow cytometric analysis of CD3+ CD4+ T cells among cells isolated from lungs of the wild type (Cre-) or Ndfip1fl/fl CD4-Cre + (cKO) animals shown in Figure 1, but focused only on litter-matched Cre- and Cre+ controls (i.e. litters in which Cre- and Cre+ mice came from the same mothers). (a-b) Percentages of lung CD4+ T cells that are IL-17A+ (a) or IFNγ+ (b). (c-d) Percentages of CD44+ CD4+ cells that are IL-17A+ (c) or IFNγ+ (d). n= 4 Cre- and n=5 Cre+ in two independent experiments. *$p<0.05$, **$p<0.01$,****$p<0.0001$. p values were calculated by unpaired two-tailed T tests. All error bars represent mean +/- SEM

Supplementary Figure S2. Ndfip1 does not restrain IFNγ secretion from restimulated Th1 cells. (a) Representative plot showing Tbet+ IFNγ+ Th1 cells after Th1 polarization and IL-2 expansion of IL-4-/- or Ndfip1 IL-4 DKO CD4+ T cells. (b) Summary of IFNγ ELISAs from plated Th1 cells. Data is pooled for n=2 Ndfip1 IL-4 DKO and n=3 IL-4-/- animals and is analyzed by 2-way ANOVA. *$p<0.05$, **$p<0.01$,***$p<0.001$. Error bars represent mean +/- SEM

Supplementary Figure S3. Loss of Ndfip1 in T cells drives a spontaneous colitis in mice
(a) Colon histology of ten week old Ndfip1 fl/fl CD4 Cre+ (cKO) or WT (Ndfip1 fl/fl Cre- or Ndfip1 fl/+ Cre-) animals showing evidence of spontaneous colon inflammation in cKO animals. Bars represent 100um and inset shows enlarged version of the boxed section of the image. (b) Ndfip1 fl/fl CD4 Cre+ (cKO) and WT animals do not show differences in colon length. (c) Flow cytometry data indicating a trend towards increased CD11b+ CD11c- Ly6G+ neutrophils in the colons of Ndfip1 fl/fl CD4 Cre+ (cKO) versus WT animals. Data is shown for an n= 3 Ndfip1 fl/fl CD4 Cre + and n=3 WT (Ndfip1 fl/fl Cre- or Ndfip1 fl/+ Cre-) animals at ten weeks old. All error bars represent mean +/- SEM

Supplementary Figure S4. Th17 cells lacking Ndfip1 show a gene expression profile associated with Th17 pathogenicity.
Naïve sorted CD4+ T cells were differentiated into Th17 cells for 5 days. Cells were harvested and expanded in IL-2 for 3 days and then used for qPCR gene expression analysis shown here and also used in the ELISA studies in Figure 4. Data is shown for n=4 mice per genotype in 2 independent differentiation experiments. All error bars represent mean +/- SEM
